# Role of IL33 in chronic inflammation and microvascular damage as a reflection of organ damage on a cohort of patients with acromegaly

**DOI:** 10.1007/s40618-024-02305-6

**Published:** 2024-02-08

**Authors:** D. Costa, C. Pellicano, V. Mercuri, E. D’Ascanio, G. Buglione, G. Cicolani, U. Basile, G. Leodori, P. Gargiulo, E. Rosato

**Affiliations:** 1 Department of Experimental Medicine, Endocrinology-Pituitary Disease, “Sapienza” University of Rome, Rome, Italy; 2 Department of Traslational and Precision Medicine, Sapienza-University of Rome, Rome, Italy; 3 Laboratory of Seminology-Sperm Bank “Loredana Gandini”, Department of Experimental Medicine, “Sapienza”, University of Rome, Rome, Italy; 4 UOC of Clinical Pathology DEA II Level, Hospital Santa Maria Goretti of Latina-Italy, Latina, Italy

**Keywords:** IGF-1, Acromegaly, Chronic inflammation, Interleukin 33, Endothelial disfunction

## Abstract

**Aim:**

Acromegaly is a rare chronic disease, caused by the over-secretion of growth hormone (GH), that creates a pro-inflammatory state, but the exact mechanisms by which GH or insulin-like growth factor 1 (IGF-1) act on inflammatory cells are not fully understood. Aim of the study was to evaluate Interleukin-33 (IL33) and the skin perfusion of hands in patients with acromegaly (AP) and healthy controls (HC).

**Methods:**

IL33 have been assessed in 40 AP and 40 HC. IL 33 was determined and skin perfusion of hands was assessed by laser speckle contrast analysis (LASCA) in both populations.

**Results:**

IL33 was significantly higher in AP compared to HC [45.72 pg/ml (IQR 28.74–60.86) vs 14 pg/ml (IQR 6.5535); *p* < 0.05]. At LASCA, peripheral blood perfusion (PBP) was significantly lower in AP compared to HC [53.39 pU (IQR 40.94–65.44) vs 87 pU (IQR 80–98) *p* < 0.001]. The median values of ROI1, ROI2 and ROI3 were significantly lower in AP compared to HC [97.32 pU (IQR 50.89–121.69) vs 131 pU (IQR 108–135); *p* < 0.001], [58.68 pU (IQR 37.72–84.92) vs 83 pU (IQR 70–89), *p* < 0.05] and HC [52.16 (34.47–73.78) vs 85 (78–98), *p* < 0.001], respectively. The proximal–distal gradient (PDG) was observed in 18 of 40 (45%) AP.

**Conclusion:**

Serum IL33 is higher in AP compared to HC; conversely a reduction of PBP of hands was present in AP compared to HC, probably due to endothelial dysfunction, strictly dependent on acromegaly and are not influenced by the choice of treatment.

## Introduction

In Acromegaly the over secretion of growth hormone (GH) stimulates the endogenous production of insulin-like growth factor 1 (IGF-1), primarily by the liver, however, several other cells, including immune cells, are involved [1–4]. Although the exact mechanisms by which GH and/or IGF-1 could affect inflammatory cells are not fully understood, recent studies confirmed a chronic proinflammatory milieu in acromegalic patients [5–10].

Over the last few years, there has been a particular and growing interest in a new order of pro-inflammatory mediator that seem to play a pivotal role in different phases of inflammation such as Interleukin-33 (IL33)**.** IL33 is a member of the Interleukin-1 (IL1) family of cytokines highly expressed by endothelial cells (EC). During acute inflammation it acts as “alarmin” and it is released in damaged tissue to modulate proinflammatory responses. Its receptor, the suppression of tumorigenity receptor (ST2L) is expressed on most immune cells and plays an indirect role in the pathophysiology of several pro-inflammatory and autoimmune diseases [11, 12].

IGF-1 excess is also involved in microvascular inflammation leading to endothelial damage [13–15], both directly and indirectly due to the development of comorbidities that interfere with the microcirculation such as hypertension, diabetes and obstructive sleep apnea syndrome (OSAS) [16, 17].

Laser speckle contrast analysis (LASCA) is a non-invasive diagnostic tool for evaluating skin perfusion and studying subclinical microvascular dysfunction, Proximal–distal gradient (PDG) can identify subclinical microangiopathic impairment [18]. In normal condition, perfusion skin of the distal part of the hand (fingers) is more than proximal ones. The proximal–distal gradient (PDG) is present when the perfusion mean difference between the distal finger area and the proximal finger area was > 30 pU. Value of 30 pU was chosen as the limit between normal and abnormal perfusion [19].

In the literature, there are no studies relating to the impact of acromegaly therapies on inflammation and microcirculation. Somatostatin analogues (SSAs) interfere with the effects of GH and IGF-1, amplified by concomitant hyperinsulinism, in particular on atherosclerosis. About growth hormone–receptor antagonist (Pegvisomant) there are only a few interesting studies on a controversial immunomodulatory effect [20–22].

The aim of the study was to evaluate the presence of microvascular damage and any correlation between the therapy for the underlying pathology and the chronic inflammatory and/or microvascular damage on a cohort of acromegalic patients.

## Materials and methods

Forty acromegalic patients (AP) followed regularly in the Center for the Management of Pituitary Diseases—Department of Experimental Medicine Endocrinology-Sapienza University of Rome were enrolled in this study. Forty Healthy Controls (HC) matched for sex, age and body mass index (BMI) were also enrolled. HC were recruited among health workers of the Department of Translational and Precision Medicine of Sapienza University of Rome.

### Inclusion and exclusion criteria

Inclusion criteria for AP were: confirmed diagnosis of acromegaly, age ≥ 18 years and ≤ 65 years, written informed consent or equivalent document (e.g. written information) based on local regulations obtained prior to any data collection activity. Inclusion criteria for HC were: age ≥ 18 years and ≤ 65 years and written informed consent or equivalent document (e.g. written information) based on local regulations obtained prior to any data collection activity. Exclusion criteria, for both AP and HC, were age < 18 years or age > 65 years, diagnosis of organ-specific or systemic autoimmune disease, solid or hematologic malignancy, therapy with cortisone acetate, l-thyroxine replacement therapy not well-controlled, diabetes or impaired glucose tolerance, dyslipidemia, pregnancy, breastfeeding and smoke.

### Clinical assessment

In all AP, the diagnosis was performed according to the current guidelines between 1983 and 2021 [23]. The diagnosis of acromegaly was made in the presence of IGF-1 levels above the upper limit of normal range (ULNR) and by lack of suppression of GH to < 1.0 μg/l during an oral glucose load test (2 h after 75 g of oral glucose). The disease control was defined in the presence of IGF-1 levels below the ULNR and a GH level < 1.0 μg/l [23]. After a biochemical diagnosis of acromegaly, was performed an imaging study with magnetic resonance imaging (MRI) to visualize tumor size and appearance. Disease duration was considered as the time elapsed from the appearance of signs and symptoms of the disease to the moment of inclusion in the study. AP were grouped according to medical or surgical therapy. Three patients were newly diagnosed and therapy naïve, 15 treated with somatostatin analogues (SSA) with or without previous surgical treatment, 9 treated with GH receptor agonist (Pegvisomant) with or without previous surgical treatment,13 treated only with surgical treatment. Disease control is defined by IGF-1 levels based on age, expressed in ng/mL, and on GH levels. All AP and HC enrolled in this study had no symptoms or signs of systemic autoimmune disease.

### Laboratory assays

The serum values of GH and IGF-1 were performed according to the guidelines [23]. In all AP and HC, antinuclear antibodies (ANA), antibodies to citrullinated protein (Anti-CCP), C-reactive protein (CRP), erythrocyte sedimentation rate (ESR) were evaluated to exclude systemic autoimmune diseases. Complement fractions C3 and C4 were dosed by turbidimetry. Antibody profile was evaluated by indirect immunofluorescence (IFI) for the detection of ANA and definition of the immunofluorescence pattern (homogeneous, speckled, centromeric, cytoplasmic) [24], and a commercial ELISA kit were used for the detection of anti-CCP and CRP. Due to the negativity of ANA it was not necessary to carry out the evaluation of the extractable nuclear antigen (ENA). Automated method was used for ESR assessment. IL33 was determined by commercial competitive ELISA kit (MyBiosource), with a sensitivity of 1 pg/ml and an assay range of 0–1000 pg/ml. Peripheral venous blood samples have been acquired in tubes not containing sodium citrate or EDTA and remained at room temperature for 2 h to allow clotting; then the samples were centrifuged at 1000×*g* for 15 min. The serum samples have been transferred to 1.5 ml Eppendorf and stored at− 80 °C until the time of assay, according to the instructions provided by the manufacturer.

### Microvascular assessment

Peripheral blood perfusion (PBP) of the hands was analyzed by LASCA (Pericam PIM 3, Perimed, Jarfalla-Stockholm, Sweden). The scanner was perpendicular to the hand with a distance of 15 cm, according to the manufacturer’s instructions. Two-dimensional images (measurement area 12 × 12 cm) were acquired at the greatest time and spatial resolution. One hand was imaged at a time. The final result was an average of the two hands. PBP was expressed as arbitrary perfusion units (pUs). Based on our previous study, the dorsum of the hand was divided into three regions of interest (ROI): ROI1, ROI2 and ROI3. ROI1 included the three fingers of the hand from the second to the fourth distal to the proximal interphalangeal finger joint [25]. ROI2 included the area between the proximal interphalangeal finger joint and the metacarpophalangeal joint [25]. ROI3 included only the dorsum of the hand [25]. PDG was identified when the perfusion mean difference between ROI1 and ROI2 was > 30 pU. All values were calculated as the mean of both hands.

### Statistical analysis

SPSS version 26.0 software was used for statistical analysis. The coefficient of skewness and kurtosis by the Shapiro–Wilk test was used to evaluate the normal distribution of data. All results are expressed as mean and standard deviation (SD) or median and interquartile range (IQR), as appropriate. Group comparisons were made by Student’s unpaired 2-tailed *t*-test or Mann–Whitney test, as appropriate. Pearson product-moment correlation coefficient or Spearman’s rank correlation coefficient, as appropriate, were used to test for associations between numerical variables. The chi-square test or Fisher’s exact test, as appropriate, was used to compare categorical variables. *p* values < 0.05 were considered significant.

## Results

The median age of patients was 55 years (IQR 44–65 years) and the disease duration was 15 years (IQR 12–25.5 years). Most patients (37/40, 92.5%) received treatment for acromegaly: neurosurgery, radiotherapy, drug therapy, alone or in combination. Three patients were newly diagnosed and therapy naïve; 15/40 treated with somatostatin analogues (SSA), in 11/15 cases preceded by NCH; 9 under treatment with growth hormone agonist (Pegvisomant), in 6/9 cases preceded by NCH; 13/40 patients had been radically treated with surgical therapy alone. A total of 40 HCs matched for gender (*F* = 27, 67.5%), age (median 54.4 years and IQR 44–65) and BMI 24.1 kg/m^2^ were enrolled in this study. All AP and HC were negative for ANA, anti-CCP and Reuma test. Clinical and anamnestic data are reported in Table 1**.**

**Table 1:** Clinical and anamnestic data of patients with acromegaly(AP)

Age, years, median and IQR	55 (44–65)
Female, *n* (%)	25 (62.5)
Age at diagnosis, years, median and IQR	39 (33–44.5)
Duration of disease, years, median and IQR	15 (12–25.5)
Diagnostic latency, years median and IQR	2 (1–5)
IGF-1, ng/ml, median and IQR	216 (167–321)
Target, *n* (%)	25 (62.5)
Hypertension, *n* (%)	27 (67.5)
OSAS, *n* (%)	16 (40)

*IQR* interquartile range, *IGF-1* insulin-like growth factor 1

### IL33

**Fig. 1:**
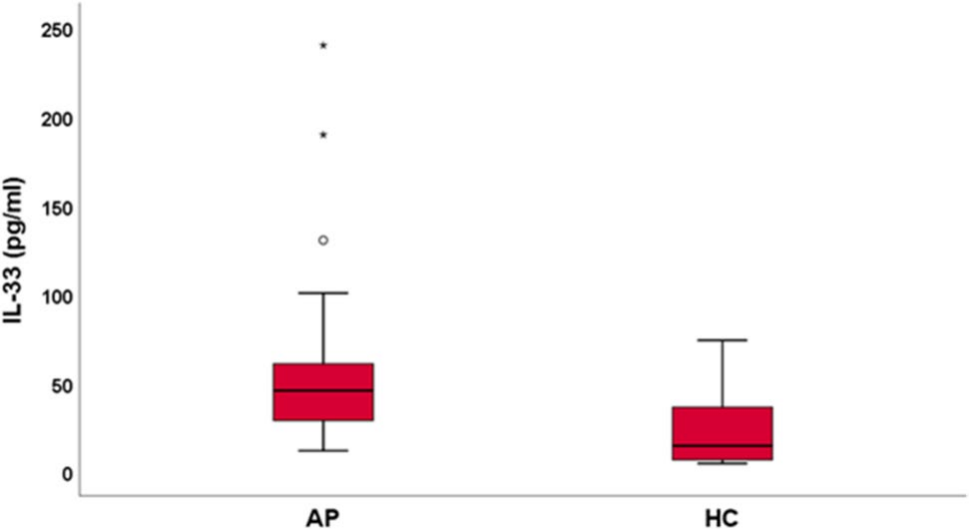
IL33 values in acromegalic patients (AP) and healthy controls (HC)

The median values for IL33 were 45.72 (28.74–60.86) pg/ml, significantly higher in APs compared to HCs: 14 (IQR 6.55–36.35) pg/ml; (*p* < 0.05) (Fig. 1).

### Perfusion

The median values of PBP and in ROI1, ROI2, ROI3 were, respectively: 53.39 (40.94–65.44) pU, 97.32 (50.89–121.69) pU, 58.68 (37.72–84.92) pU, 52.16 (34.47–73.78) pU. The proximal–distal gradient (PDG) was found in 18 (45%) acromegalic patients (AP).

Table 2 shows the median reference values for the LASCA parameters.

**Table 2:** Hand perfusion in acromegalic patients (AP) and healthy controls (HC)

	AP	HC	*p*
PBP, pU, median and IQR	53.39 (40.94–65.44)	87 (80–98)	< 0.001
ROI1, pU, median and IQR	97.32 (50.89–121.69)	131 (108–135)	< 0.001
ROI2, pU, median and IQR	58.68 (37.72–84.92)	83 (70–89)	< 0.005
ROI3, pU, median and IQR	52.16 (34.47–73.78)	85 (78–98)	< 0.001
PDG, *n* (%)	18 (45)	40 (100)	< 0.001

*PBP* peripheral blood perfusion, *ROI* region of interest, *PDG* proximal–distal gradient

**Fig. 2:**
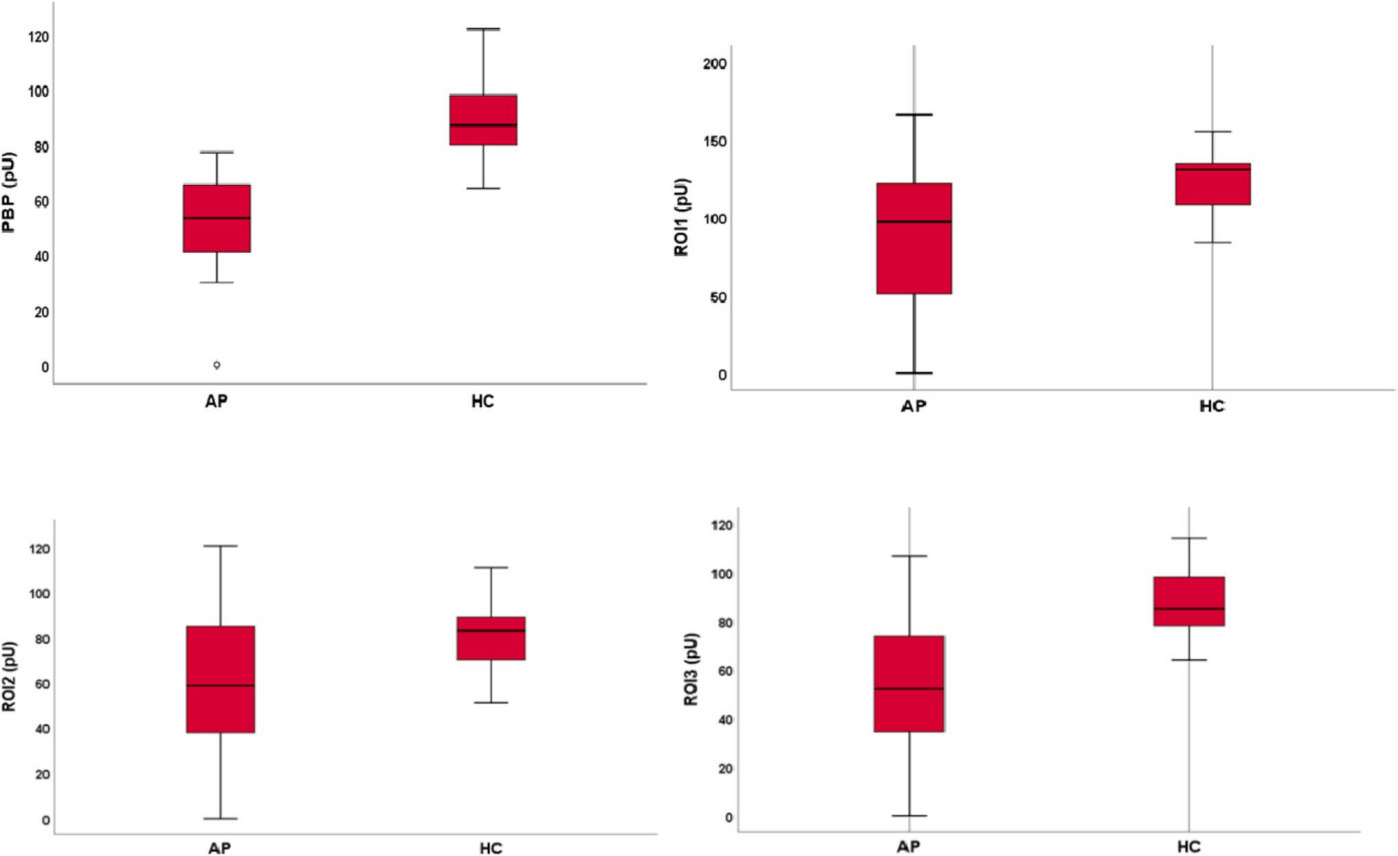
Perfusion values of acromegalic patients (AP) and healthy controls (HC)

In HCs, the median PBP value was 87 (80–98) pU with PDG present in 40 (100%) subjects. PBP, ROI 1, ROI2 and ROI 3 were significantly higher in HC compared to AP (*p* < 0.001; *p* < 0.001; *p* < 0.005; *p* < 0.001, respectively) (Fig. 2).

No significant correlation was found between IL-33, LASCA parameters, and the biochemical control of the disease (Table 3).

**Table 3:** Correlation IL33, LASCA parameters and biochemical control of disease (Target)

Variables	Target yes	Target no	*p*
IL-33, pg/ml	46.5 (34.6–62.1)	45.3 (27.9–60.9)	> 0.05
PBP, pU	49.8 (40.4–66.5)	54.5 (45.5–61.1)	> 0.05
ROI1, pU	92.8 (41.8–120.7)	100.7 (81.8–123.1)	> 0.05
ROI2, pU	58.4 (32.4–81.2)	67.6 (44.3–84.9)	> 0.05
ROI3, pU	52.4 (34.7–75.3)	47.7 (36.1–71.8)	> 0.05
PDG, pU	31.5 (4.7–42.9)	30.2 (14.4–42.8)	> 0.05

All results are expressed as median and interquartile range (IQR)

### Comorbidity

The correlation between IL 33 and the LASCA parameters did not highlight any significance. The presence of comorbidities (hypertension, OSAS of any grade) does not correlate with IL 33 levels, nor with LASCA parameters, except for the statistically significant correlation between the presence of OSAS of any grade and PDG (*p* < 0.01).

### Treatment

**Fig. 3:**
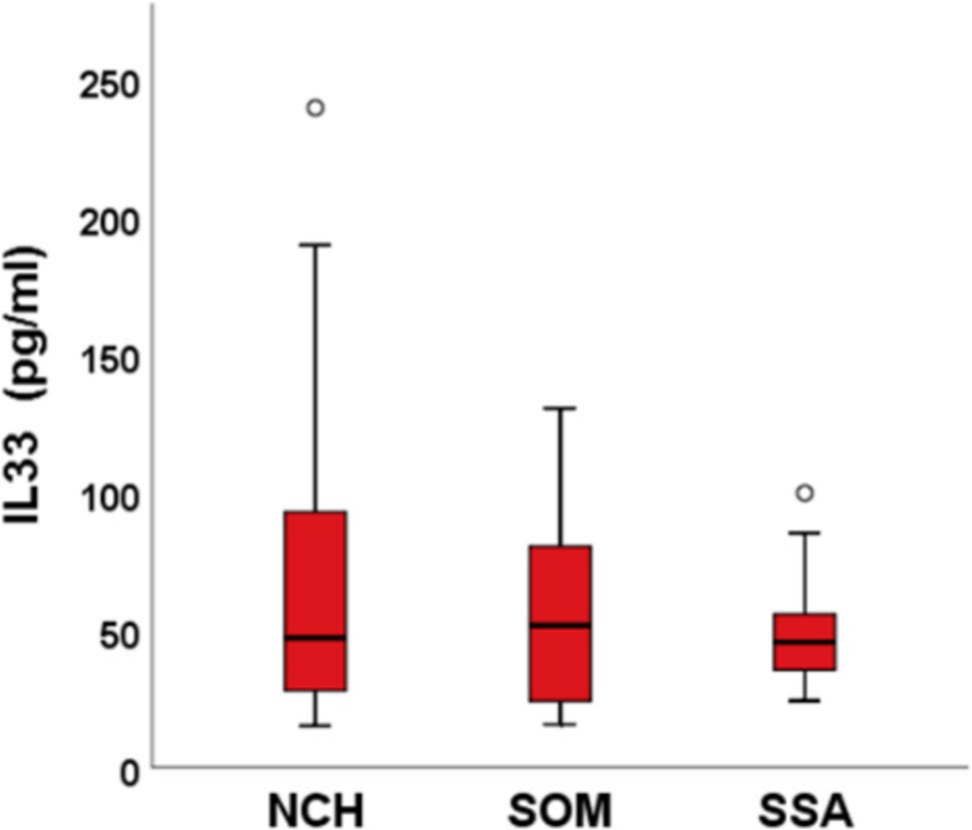
IL33 values in different treatments with Somatostatin Analogues (SSA), Pegvisomant (SOM) and Neurosurgery (NCH)

**Fig. 4:**
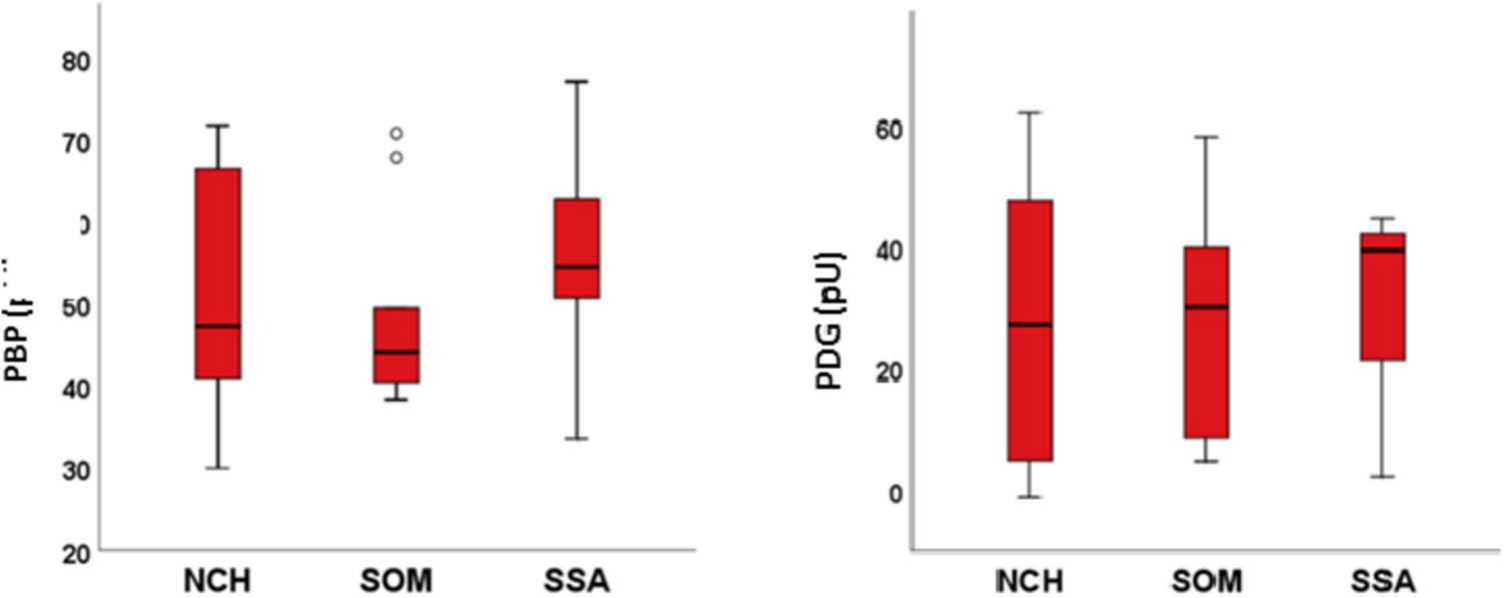
Peripheral perfusion (PBP) and proximal–distal perfusion gradient (PBG) values in different treatments with Somatostatin Analogues (SSA), Pegvisomant (SOM) and Neurosurgery (NCH)

Treatment with SSA, Pegvisomant or neurosurgery does not correlate with the clinical, biochemical (Fig. 3) and instrumental parameters examined (Fig. 4).

## Discussion

Supported by the data in the literature relating to the ability of GH and IGF-1 to stimulate the cells of the innate and adaptive immunity and the consequent production of cytokines, we previously started a pilot study to evaluate the microvascular profile and proinflammatory state of patients with acromegaly through the analysis of a marker that has recently acquired interest due to its important role in triggering a proinflammatory state: IL33.

Recent studies have demonstrated that IL33 promotes angiogenesis and vascular permeability in vitro and in vivo, evident phenomenon in the context of the inflammatory process [26–28]. It could contribute to the development of cardiovascular disease (CVD), but further clinical studies are needed to determine its real implication.

Our previous study on 20 patients with acromegaly showed that IL33 is higher in patients with acromegaly compared to healthy controls. It should be noted that these inflammatory characteristics of the AS patient must be defined as subclinical since the common biochemical markers of inflammation in clinical practice as ESR and CRP were within normal ranges [29].

The same data were obtained in this study on a larger sample of patients (40), confirming that in patients with acromegaly the inflammation is persistent due to the increase in the proinflammatory IL33, independently of the biochemical control of the disease. These data are also in line with the literature as also supported by Wolters et al. who observed chronic inflammation in acromegaly patients both controlled and uncontrolled [30].

Furthermore, we demonstrated that PDG is absent in 55% of patients with acromegaly. In LASCA, reduction of PBP with the absence of PDG is associated with functional dysfunction of the microcirculation of the hands. Maison et al. demonstrated that the ability of vascular smooth cells to produce cutaneous vasodilation is normal, but endothelium-dependent vasodilation is impaired. Furthermore, the sympathetically mediated vasoconstrictor response is increased in patients with acromegaly [31]. Paisley et al. also demonstrated that active acromegaly is associated with hypertrophic remodeling of the vascular wall and impaired endothelial function due to the reduction of nitric oxide and endothelium-derived [32]. We can assume that endothelial dysfunction plays a key role in the reduction of PBP and the absence of PDG. This endothelial dysfunction may contribute to hypertension and represent a risk factor for cardiovascular complications in acromegaly.

Further relevant data have been obtained with the LASCA. Peripheral blood perfusion (PBP) and Region of Interest (ROI) 1, 2, and 3 were significantly lower in AP than HC. From the comorbidity analysis of our current study turned out that patients with OSAS present a higher PDG.

This observation suggests a question related to pulmonary perfusion: can the reduced skin perfusion be considered a mirror of a similar pulmonary condition? This is not yet clear. A study on systemic sclerosis (SSc) have not found statistically significant correlation between the PBP or both diffusing capacity of the lungs for carbon monoxide DLCO and Pulmonary Arterial Hypertension (PAH) [33]. In our previous study on pulmonary perfusion. we showed a positive correlation between baseline serum IGF-1 levels and DLCO/VA [34].

In light of our results, neoangiogenesis, especially an increase in alveolar capillarization, as a response to inflammation, alterations of perfusion and hypoxia, could thus justify the increase in DLCO in acromegalic patients with OSAS. The same compensation mechanism could also explain the fact that in our acromegalic patients with OSAS the PDG (proximo-distal gradient) is greater than in patients who do not suffer from this comorbidity.

Therefore, the state of the microcirculation at the level of the fingers could represent an indirect estimate of the state of the microcirculation at the organ level.

Finally, it is not known in the literature whether treatments for acromegaly can play a role as modulators of the inflammatory process.

There are few interesting studies in the literature on the controversial immunomodulatory effect of Pegvisomant. The thymotropic properties of the GH/IGF-1 somatotropic axis imply an interaction between GH and exogenous Growth Hormone receptor (GHr) expressed by the epithelial cells of the thymus. In particular, GH downregulates the expression of GHr, with a parallel decrease of thymic IGF-1. These effects are only partially reversed by Pegvisomant [21]. Other studies suggest that the altered complex immunological fingerprint and signs of endothelial damage of AP are only partially normalized by disease-specific treatment [14, 22].

In any case, our study did not highlight any possible correlation between treatment for Acromegaly (SSA, SOM or NCH) and inflammatory and microvascular aspects. This demonstrates that inflammation and microvascular damage are strictly dependent on the underlying pathology and are not influenced by the choice of treatment.

The biases of the previous study relating to the analysis of comorbidities and treatment of Acromegaly have been filled in the current project.

## Conclusion

The patient with acromegaly presents a chronic inflammatory state whose pathogenesis is not yet completely known and the investigation of new inflammatory factors could have an important impact on the therapeutic target.

Furthermore, the possibility of having easy-to-perform, non-invasive methods available, capable of evaluating the integrity of the microcirculation at the level of the periungual capillaries of the fingers of the hand, allows us to indirectly evaluate the state of various organs and systems, otherwise studied through methods invasive, including the heart, lungs and kidneys, whose alterations can be responsible for important pathological conditions for patients.
